# A global and local perspective of interruption frequency in a visual search task

**DOI:** 10.3389/fpsyg.2022.951048

**Published:** 2022-09-16

**Authors:** Tara Radović, Tobias Rieger, Dietrich Manzey

**Affiliations:** Department of Psychology and Ergonomics, Engineering, and Organizational Psychology, Technische Universität Berlin, Berlin, Germany

**Keywords:** visual search, interruptions, performance, goal activation, task resumption

## Abstract

We investigated the impact of frequency of interruptions in a simulated medical visual search task. Participants (*N* = 150) performed the visual search task during which they were interrupted by a number-classification task in 25, 50, or 75% of all trials, respectively, reflecting the frequency conditions (i.e., low, mid, high). Target presence (i.e., present vs. absent) and interruption (i.e., uninterrupted vs. interrupted) were varied within-subjects, and interruption frequency was varied between-subjects. Globally, on a frequency condition level, participants in the low frequency condition had longer mean response times (RT) for the primary visual search task than in the high condition, but there were no other performance differences between the three frequency conditions. Locally, on the level of specific interruption effects, accuracy decreased directly after interruptions for target present but not for target absent trials. Furthermore, interruptions caused significant interruption costs, reflected in slower overall RTs in interrupted than in uninterrupted trials. The combined findings show that especially for critical visual search tasks as in the medical field, interruptions—regardless of frequency—should be avoided.

## Introduction

Interruptions of the workflow are an unavoidable factor in everyday life. In very dynamic working fields, e.g., intensive care units or emergency medicine, medical staff get interrupted up to 23 times per hour (e.g., Grundgeiger and Sanderson, 2009; Ratwani et al., 2016; Drews et al., 2019). The performance consequences of interruptions and their frequency can be considered on what we refer to as a local and global level. The local level involves direct performance consequences on the primary task after an interruption. The global level involves more general effects of cognitive effort associated with coping with more or fewer interruptions on overall performance in terms of work speed or error proneness, which could be associated with different overall interruption frequencies. Thus far, most of the interruption research has focused on the local effects. This research has shown that interruptions have detrimental effects on the performance in the interrupted (primary) tasks. Specifically, interruptions often lead to forgetting to resume the primary task after an interruption completely (e.g., Einstein et al., 2003; Dodhia and Dismukes, 2009; Grundgeiger et al., 2010a). Besides that, if the intention of resuming the primary task after an interruption is not completely forgotten, this resumption process comes at cost and it is often related to the increased response times and increased probability of committing an error upon resumption (e.g., Hodgetts and Jones, 2006; Grundgeiger et al., 2010b; Westbrook et al., 2010; Altmann et al., 2014; Radović and Manzey, 2019, 2022).

These adverse effects of interruptions and the underlying cognitive processes are typically explained within the memory for goals theory proposed by Altmann and Trafton (2002). The theory states that cognitive tasks are goal-directed, meaning that a goal in working memory has to be activated above a certain threshold (i.e., interference level) for executing a specific cognitive task. For memorizing goals and increasing their activation above the interference level, (i.e., mean activation of other irrelevant goals in working memory) active processes, such as rehearsal, are necessary. When the goal is not relevant anymore, e.g., after the task is executed or in the case of interruption, its activation will exponentially decay with time. The re-activation of the primary task goals can be supported by using internal or external cues, and, if it still remains below the interference level, there is an increased probability of committing an error in the primary task after an interruption.

However, while this theory provides a straightforward explanation for performance consequences of single interruptions on the primary task, predictions are less clear with respect to effects of different frequencies of interruptions. It could be assumed that more frequent interruptions would increase the number of irrelevant goals in working memory, leading to a generally higher interference level one needs to overcome when resuming the primary task after a given interruption. Consequently, frequent interruptions might be assumed to further raise resumption times and error rates after an interruption compared to the condition in which interruptions occur less often. However, such an explanation could only partially account for results obtained in previous research. For example, while Speier et al. (1999) as well as Westbrook et al. (2010) provided evidence in line with predictions derived from memory for goals theory, the findings of Monk (2004) seem to be in contrast to that. He found significantly faster and somewhat more accurate performance after an interruption when interruptions occurred with a higher compared to a lower frequency. Thus, while the previous research has provided a fairly consistent pattern of consequences of single interruptions on primary task performance, the effects of different interruption frequencies are less clear, thus far, with only very few studies providing conflicting results (Speier et al., 1999; Monk, 2004; Westbrook et al., 2010).

A theoretical framework dealing with global effects of interruptions, i.e., cumulative effects of interruptions on overall workload has recently been proposed by Baethge et al. (2015). Within this framework, cumulative effects of interruptions are determined by the interaction between different properties of the interruption and the interrupted tasks (e.g., complexity, length, or frequency) whose effects accumulate over time and influence the performance. For example, when a task gets interrupted very frequently, it would pose higher cognitive demand than a situation in which the same task is interrupted less often. Consequently, the two situations would lead to different cumulative effects, as they carry different cognitive loads in terms of the processing of the tasks, scheduling them in a limited time frame, and shifting between them. These demands accumulate over time, possibly trigger additional compensatory responses, and overall, they lead to accelerated increase of strain. At the same time, overall performance would follow a different trajectory over time. At first, if cognitive capacity is not already exceeded, interruptions can increase arousal to an optimal level, which is specific for a certain context. This, in turn, can result in better performance in the tasks. However, with long exposure to such a strain, it becomes difficult to maintain the same level of performance. This may lead to reduced effort and/or employing risky behaviors, resulting in degraded performance. Thus, greater cumulative effects of interruptions are related to an increase in strain, which is assumed to affect the performance in an inverted U-shape manner. This means that the overall performance in the task would not necessarily be impaired by interruptions but reaches its maximum at some optimal/mid-level frequency of interruptions in a certain situation (Baethge et al., 2015).

Thus, in contrast to the memory for goals theory, the integrative theoretical framework for the study of cumulative interruptions at work focuses on global effects of interruptions over a given time period instead of immediate effects of isolated interruptions which are analyzed locally, typically at the first step following the interruption (Altmann et al., 2014). Moreover, the theoretical framework for the study of cumulative interruptions would also predict positive effects of an increasing frequency of interruption on performance in primary tasks if this frequency remains in an optimal-mid range for a certain situation.

Note, however, that the two theoretical approaches are not mutually exclusive and could complement each other in explaining different aspects of interruption-induced performance consequences in a task. Previous studies have suggested different effects of interruptions on the local and global level (Zijlstra et al., 1999; Ratwani et al., 2006). For example, Ratwani et al. (2006) found the known detrimental effects of interruptions on a local measure of performance, i.e., performance immediately after an interruption. Yet, they also showed that on a more global level, the very same interruptions had positive effects, that is, participants generally worked faster and more accurately in the interrupted blocks than in the uninterrupted blocks. Previous research also suggests that these local and global effects of interruptions on primary-task performance can be emphasized by an increased interruption frequency. For example, in the study by Zijlstra et al. (1999), participants were interrupted one or three times per task while conducting a primary task consisting of several goals that had to be stored in memory. When participants were interrupted three times per task, more time was required for reorientation and resumption of the primary task immediately after interruption compared to the one-interruption condition. However, total time spent on the primary task execution was shorter compared to the one-interruption condition, suggesting some sort of compensation and general speeding up later in the task when interruptions occurred more frequently.

The present research aims at investigating local and global effects of interruptions on performance in a visual search task. In a visual search task, one needs to determine whether there is a certain target present (e.g., a malign structure in a mammogram) or absent (e.g., just benign tissue). In our view, due to the specific demands this type of task seems to be particularly suitable for investigating local and global effects of interruptions at the same time. Namely, after being interrupted in a visual search task, some spatial re-orientation is required for resuming the primary task. This is specifically demanding, as cues that typically support memory of where the primary task was interrupted, are not really available in visual search tasks as visual search has very little memory with respect to previously searched areas (e.g., Horowitz and Wolfe, 1998; Võ et al., 2016; Drew and Williams, 2017). Thus, resuming a visual search task after an interruption might not only involve a re-activation of the task goal in memory, but a complete reorientation where to continue the interrupted search. Moreover, in laboratory as well as in applied visual search tasks (e.g., luggage screening) many individual trials often have to be performed one after the other (e.g., Buser et al., 2020). This task processing scheme seems well-suited to study both local and global performance consequences of interruptions. In addition, knowledge about the local and global effects and their possible interplay seems to be directly practically relevant with respect to possible performance consequences of interruptions in typical applied settings.

Interruptions in visual search tasks have been studied previously, though mainly on a local level. This research has generally confirmed the adverse effects of interruptions on the local performance in a primary task after an interruption in terms of resumption times (Williams and Drew, 2017; Drew et al., 2018; Brazzolotto and Michael, 2019, 2020) and accuracy (Borowsky et al., 2016; Wynn et al., 2018). However, it is unclear, thus far, whether these local effects of interruptions also propagate to a more global level of visual search performance. In addition, relatively little is known about the effects of frequency of interruptions on local and global level of task performance. As was mentioned above, there is some evidence in the interruptions literature (e.g., Zijlstra et al., 1999; Ratwani et al., 2006) that frequency of interruptions might impact local and global performance in primary tasks. Studying these effects in visual search also does not only appear interesting from a theoretical view but also from an applied perspective, as there are numerous real-world examples of visual search tasks which are interrupted, even at different frequencies. For example, think of luggage screeners at different airports where the work demands in terms of possible frequencies of interruptions might dynamically differ.

The specific visual search task used in our study simulated typical cognitive demands of an x-ray screening task. Our participants had to perform a given number of screening trials in a row while they were interrupted from time to time by an unrelated number classification task. Frequency of interruptions was defined as the rate of screening trials where interruptions occurred, and varied across three different groups, with low, medium and high frequency defined as 25, 50, and 75%, respectively.

The local performance consequences of these interruptions were assessed through the interruption costs, i.e., by comparing response times and accuracy rates for visual screening in trials where the screening was interrupted compared to the uninterrupted trials. In contrast, the corresponding global effects of interruptions on visual search performance were assessed by comparing the different frequency conditions on speed and accuracy of visual search at a global level which involves both interrupted and uninterrupted trials. In this way, we were able to assess the effects of low, medium, and high interruption frequency on the workflow and directly test the predictions of the theoretical framework for cumulative interruptions regarding the inverted U-shape relationship between the overall performance and the frequency of interruptions.

Locally, we expected a significant main effect of interruption presence, i.e., greater performance costs in both response time and accuracy in the interrupted trials compared to the uninterrupted ones as proposed by the memory for goals theory (Altmann and Trafton, 2002) as well as earlier research (e.g., Borowsky et al., 2016; Williams and Drew, 2017; Drew et al., 2018). The costs were expected as a result of the extra effort when resuming the primary task after an interruption in these trials. On the global level, we expected a significant main effect of interruption frequency on response time and accuracy. Specifically, we expected to find an inverted U-shape relationship between the interruption frequency and performance, with generally faster and more accurate responses in the mid-frequency than in the low and high frequency conditions. Such a finding would be in line with the predictions of the theoretical framework of Baethge et al. (2015).

Moreover, interruption presence and interruption frequency could also interact. Specifically, the local interruptions costs could increase with higher frequency which would be in line with an assumption derived from the memory for goals theory (Altmann and Trafton, 2002), as there is an increased number of irrelevant task goals interfering with the primary task. In contrast, a possible global-local interaction could also be reflected in the inverted U-shape relationship only being true in uninterrupted trials—which would be in line with some earlier findings regarding global speed-up effects (e.g., Zijlstra et al., 1999; Ratwani et al., 2006). Independent of the specific performance consequences of interruption we further expected to replicate typical effects of visual search performance, namely faster response times in target present than in target absent trials, as is predicted by virtually any visual search model with a self-terminating component (e.g., van Zandt and Townsend, 1993; Bricolo et al., 2002; Schwarz and Miller, 2016).

## Materials and methods

This study was approved by the local ethics committee at the Department of Psychology, Technische Universität Berlin, Germany. Participants provided informed consent to participate.

### Participants

Participants were recruited *via* a web portal of Technische Universität Berlin and the participant recruitment platform Prolific. They received either course credit (Technische Universität Berlin participants) or were paid 3.75 GBP *via* Prolific. 150 participants^1^ (71 female) were randomly assigned in equal numbers to the frequency and counterbalancing conditions with the larger part of the sample being recruited *via* Prolific (*n* = 116). The proportion of participants recruited from either platform in each frequency and counterbalancing condition did not differ was [χ^2^(5, *N* = 150) = 5.781, *p* = 0.328]. Participants ranged in age from 18 to 40 (*M* = 24.91, *SD* = 5.52) and were predominantly right-handed (83.3%). Additional 25 participants were also tested but excluded from any further analyses because of low accuracy in the main blocks [i.e., either below 70% for the visual search task (low: 8, mid: 5, high: 8) or below 80% for the odd/even task (low: 1, mid: 1, high: 1)]. Moreover, another additional 22 participants were also tested but excluded because their true proportion of interruptions had fallen below the presumed frequency of the next lower frequency condition (*n* = 19 in the high frequency condition with < 50% interrupted trials, *n* = 3 in the medium frequency condition with < 25% interrupted trials). After applying these exclusion criteria, 50 participants remained in each group. The true proportion of interruptions for the participants included in the main analyses was 20.6% (min: 15.6%, max: 25.0%) in the low frequency condition, 39.9% (min: 29.2%, max: 49.0%) in the mid frequency condition, and 60.5% (min: 50.0%, max: 72.9%) in the high frequency condition.

### Tasks

The primary task was a visual search task simulating a medical x-ray screening task. Specifically, participants searched for a target letter “E”’s among distractor letters “F,” embedded in noise with the power spectrum of 1/f^3^. This type of noise resembles the power spectrum of mammograms (Burgess et al., 2001). The interruption task was a number classification task (NCT). In this task, a number is presented visually in the middle of the computer screen and participants have to indicate by pressing a key on the keyboard whether the number is odd or even.

### Apparatus and stimuli

The experiment was programmed using jspsych (de Leeuw, 2014) and was run on a JATOS (Lange et al., 2015) server so that participants could individually run the experiment in their browser.

The noise for the visual search task was the same kind of noise as in previous research (e.g., Sha et al., 2018; Hebert et al., 2020). Search items (i.e., the letters F as distractor letters and the letter E as the target letter) were placed in randomly selected locations in an invisible 10 × 10 matrix to avoid overlap of the letters. On each image, there were ten letters. If there was no target present, there were just ten “F”’s and if there was a target present, nine “F”’s were presented along with one “E.” The letters were in font size 20 in white color and were initially inserted on a black background using the R package magick (Ooms, 2020) using the font type “sans.” The letters were not rotated. Subsequently, the letter images were used as the background when blending with the noise images at an opacity of 92%. Half of the stimuli contained a target and half of the stimuli contained only distractors. The images were sized 512 × 512 px. For each experimental block, new stimuli were randomly selected.

The NCT was presented using 18 pt Open Sans Arial font. Both tasks were presented vertically and horizontally centered on screen. The numbers 1, 2, 3, 4, 6, 7, 8, and 9 were used as the number stimuli. While the stimuli of the number classification (interruption) task were presented, the view to the primary task was fully blocked.

Participants were asked to respond to the visual search task using their left index and middle fingers on the “d” and “f” keys and to respond to the number task using their right index and middle fingers on the “j” and “k” keys. The “d” key was used to indicate a target absent response and the “f” key was used to indicate a target present response. Mapping the odd/even key to the index/middle finger was counterbalanced across participants in order to avoid effects of response compatibility of response fingers.

### Procedure

Participants were randomly assigned to one of the three interruption conditions (i.e., low, medium, high frequency) and instructed to respond as fast and as accurately as possible to both the primary and interruption task. The experiment consisted of five blocks of which the first three were considered practice. The first two of these blocks were essentially single task blocks. The first block included 48 trials of the visual search task without any interruptions. Half of these trials were target present trials and the other half were target absent trials. In the second block, participants completed 24 trials of the NCT. Each trial of these first two blocks started with a 1,000 ms fixation cross (a + sign in font size 60), followed by the respective stimulus (block 1: visual search stimulus, block 2: number). Participants received trial-by-trial feedback for 1,000 ms, each, which informed about whether or not they had responded correctly. In the practice block of the visual search task, the maximum duration to respond was 10 s. In the NCT practice block, the maximum duration to respond was just 1 s in order to get participants used to speeded responses to the NCT task. If participants failed to respond in time on either task, a 5 s reminder to respond as fast and as accurately was displayed, and participants subsequently had to press any response key to continue the experiment. This was done to ensure that participants always paid attention to the task, even in this online experiment.

After the two single-task practice blocks, the third practice block including 24 trials was performed, followed by the two 48 trials experimental blocks used for the actual data collection. During these blocks, the primary visual search task had to be performed but was repeatedly interrupted at the frequency assigned to the individual participant’s experimental group. Prior to these blocks, the 10th and 30th percentile of all correct responses to the visual search task in the single-task-practice block were calculated. These time markers then were used to schedule the interruptions in the interrupted trials, i.e., the interruption onset in these trials was chosen randomly between the 10th and 30th percentiles. This was done to ensure that the interruption onsets were based on individual performance and, thus, comparable across participants without being predictable at the same time. Half of the 24 visual search trials in the practice block and half of the 48 trials in each of the experimental blocks were target present trials and half of the trials being target absent trials. The trials with an interruption were randomly selected in each block, with the constraint that always the same proportion of target present/absent trials was interrupted.

**FIGURE 1:**
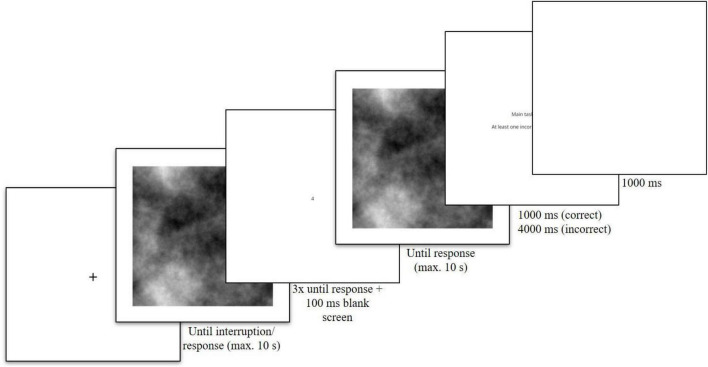
Example trial procedure with an interruption. Note that the number classification task appeared three times (i.e., required three responses). In trials without an interruption, of course only the first response screen was shown.

Despite the differences with respect to the overall number of trials, the specific trial procedure was exactly the same in the third practice and the two experimental blocks. That is, each trial started with a 1,000 ms fixation cross. Then the visual search stimulus was presented for up to 10 s or until a response was made on uninterrupted trials. On interrupted trials, the visual search stimulus was displayed for the time until the interruption onset which was drawn randomly for each trial as described above. If participants responded to the main task prior to the interruption onset, the interruption task was skipped and participants were directly shown feedback for the main task. The interruption task always consisted of three consecutive trials presented in a self-paced manner. After each response to a given number, the screen was blank for 100 ms. The NCT remained on screen until a response was made. After the three trials of the NCT were completed, the main task was shown again for a maximum of 10 s minus the time to interruption onset. After each trial of the visual search task, participants were shown feedback. If it was an interrupted trial and all responses were correct, participants were shown “Everything correct!” for 1,000 ms. If it was an interrupted trial and there was at least one incorrect response, participants were informed in which task they made an error (i.e., main task, at least one number task, or both tasks) for 4,000 ms. If the trial was not interrupted or a response was made prior to the interruption onset and the response was correct, participants were shown “Correct!” for 1,000 ms. If the trial was not interrupted or a response was made prior to the interruption onset and the response was not correct, participants were shown “Wrong!” for 4,000 ms. Feedback was followed by a 1,000 ms blank screen inter-trial-interval. The trial procedure is visualized in Figure 1.

After each block, participants were shown their mean combined overall accuracy for correct responses for the past block. Participants were also instructed that they could take a short break if they wanted along with this block feedback. The experiment took about 25 min in total.

### Design

Our experiment involved a 3 (frequency) × 2 (target vs. no target) × 2 (interruption vs. no interruption) mixed-design. Frequency of interruptions was defined as a between-subjects factor, varied to be either low (i.e., 25%), medium (i.e., 50%), or high (i.e., 75%). The other two factors represented within-subjects factors. Global effects of interruptions, as defined here, would be reflected in a main effect of frequency. In contrast, local effects were expected to be indicated by main effects of the interruption factor. Response times (RT) and accuracy in the primary task served as our dependent variables. The data are made available under the Creative Commons public domain dedication at Open Science Framework at https://osf.io/d8b59/.

## Results

### Global and local effects in the primary task

#### Total response times

The total RT for interrupted trials was calculated as a sum of the initial time passed from showing the visual search stimulus until the interruption onset and the time passed from showing the visual search stimulus again after the interruption until the key press in the primary task. For uninterrupted trials, the total RT was just the RT to the visual search task and it was measured as the time passed from showing the visual search stimulus until the response. All erroneous responses were excluded from the RT analyses. Moreover, interrupted trials where participants took more than 5,000 ms combined to respond to the three NCTs were also excluded. This resulted in a mean duration of interruptions of 2,627 ms.^2^ Finally, after visual inspection of the RT distribution, all trials with responses faster than 700 ms and slower than 7,000 ms were excluded as outliers. Applying all these exclusion criteria led to the exclusion of 1.36% of all correct trials.

**FIGURE 2:**
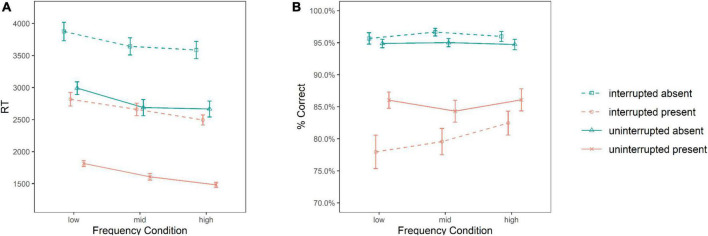
Results for mean overall response times [RT, **(A)**] and mean accuracy [% correct, **(B)**] for the main visual search task as a function of frequency condition, target presence, and interruptions. Error bars present the standard error of the mean.

We conducted a 3 (frequency) × 2 (target) × 2 (interruption) mixed ANOVA for total RTs. The corresponding means are depicted in Figure 2A. The ANOVA revealed a significant main effect of frequency condition, *F*(2, 147) = 3.298, *p* = 0.040, η*_*p*_*^2^ = 0.043, indicating a global effect of interruptions on response times in interrupted and uninterrupted trials.^3^ Pairwise comparisons with Bonferroni-corrected *p*-values revealed a significant difference between the low frequency (2,874 ms) and the high frequency (2,556 ms) conditions (*p* = 0.041), indicating a global speed-up of responses with an increasing frequency of interruptions. No pairwise comparison including the mid frequency condition (2,649 ms) was significant, though. The main effect of target presence was also significant, *F*(1, 147) = 533.289, *p* < 0.001, η*_*p*_*^2^ = 0.784. Unsurprisingly, responses were much faster in target present (2,145 ms) than in target absent trials (3,241 ms), reflecting the expected outcome of a self-terminating visual search after target detection. Moreover, there was also a significant main effect of interruption, *F*(1, 147) = 722.811, *p* < 0.001, η*_*p*_*^2^ = 0.831, with faster responses in uninterrupted (2,208 ms) than in interrupted (3,179 ms) trials, indicating the expected local effect of interruptions on primary task performance, with a mean cost effect of resuming the visual search task after an interruption of 971 ms. Finally, there was also an interaction of target and interruption, *F*(1, 147) = 7.189, *p* = 0.008, η*_*p*_*^2^ = 0.047. That is, for target present trials, the difference between uninterrupted and interrupted trials and, thus, interruption costs were larger (interrupted present: 2,656 ms, uninterrupted present: 1,634 ms, difference: 1,022 ms) than for target absent trials (interrupted absent: 3,701 ms, uninterrupted absent: 2,781 ms, difference: 920 ms). No other effect was significant (*p*s > 0.628). To gain a better understanding of the null-effects, we used the methods described in Wagenmakers (2007), with all null-effects in this ANOVA favoring the null-model over the alternative model (Pr_*BIC*_(H_0_| D) > 0.884).^4^

#### Overall accuracy

We conducted a parallel ANOVA for the percentage of correct responses to the main visual search task, and the results are visualized in Figure 2B. This ANOVA revealed a significant main effect of target, *F*(1, 147) = 201.691, *p* < 0.001, η*_*p*_*^2^ = 0.578, with more accurate responses to target absent (95.5%) than to target present (82.7%) trials. Moreover, there was a significant main effect of interruption, *F*(1, 147) = 11.756, *p* < 0.001, η*_*p*_*^2^ = 0.074, with more accurate responses in uninterrupted (90.2%) than in interrupted (88.0%) trials. However, this latter effect was modulated by a significant interaction of interruption and target, *F*(1, 147) = 24.634, *p* < 0.001, η*_*p*_*^2^ = 0.144. That is, the negative effect of interruptions was only apparent for target present trials (-5.0% interrupted vs. uninterrupted) but not for target absent trials (+ 1.2% interrupted vs. uninterrupted). The ANOVA did not reveal any other significant effects (*p*s > 0.221), including no significant global effect of interruption frequency on accuracy in interrupted and uninterrupted trials. Again, we used Wagenmakers (2007) BIC approximation of the posterior probability to better understand the null-effects. For all non-significant effects in the ANOVA, the null-model was favored over the alternative model Pr_*BIC*_(H_0_| D) > 0.724).

## Discussion

The aim of the present study was to examine local and global effects of interruption frequency in a visual search task. Locally, we found negative effects of interruptions for both speed and accuracy. Globally we found generally faster response time in the high frequency condition compared to the low frequency condition. However, the global frequency of interruptions did not interact with the local interruption performance effect.

### Implications for local and global effects of interruption frequency

Regarding local effects of interruptions, in line with the previous research (e.g., Altmann et al., 2014), standard effects of interruption presence emerged, that is, participants needed more time to respond in the primary task after the interruption and they made more errors in this case, compared to a situation in which no interruption was present. Moreover, we expected longer response times and worse accuracy with greater interruption frequency. This expectation was based on the memory for goals theory (Altmann and Trafton, 2002) and the assumption that more frequent interruptions would cause greater interference with the goal of the primary task. In this case, more time would be needed to overcome the interference and to re-activate the primary task goal sufficiently in order to resume the task after the interruption. However, this hypothesis was not supported by the data in our study, as the interruption effect was independent of interruption frequency. This finding stands in contrast with the results obtained in the previous research (Speier et al., 1999; Zijlstra et al., 1999; Monk, 2004). It seems possible that repetitive activation and re-activation of a single goal of the visual search primary task in our study increased the goal activation sufficiently, so that the different interruption frequencies did not modulate the performance in the primary task after interruption. However, as primary tasks administered in the previous studies involved more complex tasks consisting of several steps, it might be more difficult to re-activate the correct subgoal upon resumption in that case. Yet another reason could be that with increasing interruption frequency, participants were more practiced in resuming the primary task after an interruption. Both these aspects might have contributed to the fact that we did not find a global-local interaction effect.

Regarding global effects of the frequency of interruptions, we expected overall reduced response times and better accuracy in a medium frequency condition, i.e., when participants were interrupted 50% of time, compared to the low and high frequency conditions. This hypothesis was formulated based on the theoretical framework of the cumulative interruptions (Baethge et al., 2015) which predicts an inverted U-shape relationship between the interruption frequency and overall performance. Assuming that a medium frequency condition would pose an optimal cognitive demand and increase arousal and vigilance in the task, better performance in this condition compared to the low and the high frequency conditions was expected. Admittedly, this hypothesis was somewhat speculative, as an optimal interruption frequency for a certain primary task is difficult to approximate in advance. However, the results showed a rather linear trend toward faster response times with more frequent interruptions. Specifically, response times were descriptively slower in the low frequency than in the medium frequency condition and significantly slower than in the high frequency condition. This finding is generally in line with results of the previous research which have also shown that increased interruption frequency can have positive effects on global measures of performance (e.g., Zijlstra et al., 1999; Ratwani et al., 2006; Mark et al., 2008) leading to speeded performance in the primary task. No significant effect of interruption frequency on accuracy was found, though.

### Implications for interrupted visual search

Standard effects of target presence were replicated, as participants were faster when a target was present compared to the situation when it was absent (e.g., Wolfe, 1998). Moreover, participants also tended to respond target absent, resulting in a higher accuracy in target absent than in target present trials, which is in line with prior research where under many conditions (and particularly with larger display size), false alarms are rare (e.g., Bricolo et al., 2002; Wolfe, 2007; Wolfe and Van Wert, 2010).

In the RTs, target presence and interruption interacted, with larger interruption costs for target present than for target absent trials. Here, the interruption-induced slowing was still true for both target present and absent trials. Most interestingly, the accuracy for target present trials further decreased in interrupted trials. That is, target present accuracy decreased (i.e., even fewer targets were detected after an interruption), whereas the accuracy on target absent trials even descriptively increased a bit. This overall pattern shows that participants tended to respond more “target absent” after interruptions. These combined findings seem to be particularly interesting from both, a theoretical as well as a practical standpoint.

It is interesting that it seems that participants had to re-start their visual search more or less completely after interruptions. This means that they likely searched through some of the same areas again after resuming the primary task subsequent to the interruption. A similar effect also has been described in earlier research (e.g., Williams and Drew, 2017). Participants were aware to have done some search before the interruption, however, this previous search was apparently of little to no use for them after the interruption. This could then have affected the termination of the search in interrupted trials (i.e., a termination after a less thorough search after an interruption). That is, participants may have thought that they have already searched both before and after the interruption, still not having found a target, deeming more trials (erroneously) as target absent. This explanation is in line with the finding of an earlier termination of the search in RTs when resuming the primary task. This interpretation is in accordance with the background that memory plays, at best, a very limited role in visual search (e.g., Horowitz and Wolfe, 1998; Peterson et al., 2001; Võ et al., 2016; Drew and Williams, 2017).

From a practical standpoint, the implication of the local effects of interruptions seems straightforward: critical visual search tasks (such as medical visual search tasks or luggage screening at airports) should not be interrupted. This is particularly important as our findings implicate that interruptions do not only slow decisions, but also make them less accurate. That is, one would certainly want to avoid a radiologist or luggage screener to miss a crucial target because they were interrupted.

### Limitations and future directions

Limitations of the present study include typical limitations of an online study. Participants performed the experiment outside of the laboratory using various computers. However, this could have reduced the effects which would have been obtained in a more controlled setting. The visual search task used in the current study is an abstract psychological task assumed to pose similar processing demands as various real-life tasks in everyday context and working environments. Finally, the consequences of committing an error in our study are not comparable to the tasks conducted in real life. The future research should consolidate the present findings in specific populations of participants and more representative tasks for specific working environments.

## Conclusion

To the best of our knowledge, this study is among the first attempts to examine local and global effects of interruption frequency on the performance in a visual search task. The results provide evidence for a global speed up in the visual search task with increasing frequency of interruptions. Moreover, it seems that interruptions specifically increase the probability of missing a target when resuming the visual search task after the interruption, which represents a clear security risk in a plethora of work domains, such as medicine.

## Data availability statement

The datasets presented in this study can be found in online repositories. The names of the repository/repositories and accession number(s) can be found below: https://osf.io/d8b59/.

## Ethics statement

The studies involving human participants were reviewed and approved by the Ethics committee at the Department of Psychology and Ergonomics, TU Berlin. The patients/participants provided their written informed consent to participate in this study.

## Author contributions

TRa and TRi performed the material preparation, data collection, and analysis. TRa and TRi wrote and draft the manuscript. All authors commented on previous versions of the manuscript, read and approved the final manuscript, and contributed to the study conception and design.

## Footnotes

1 Sample size of 51 participants was calculated using the Gpower software (Faul et al., 2007) for a 3 × 2 mixed design, that is, the most crucial potential interaction for global and local effects, for observing η*_*p*_*^2^ = 0.05 with power of 0.80, alpha level of 0.05. To also be able to detect the main effect of frequency condition (between-subjects), we ran an additional power analysis for observing η*_*p*_*^2^ = 0.05 with the goal of power of 0.80. This analysis resulted in a required total sample size of 153 participants.

2 No significant effect of frequency condition, target presence in the primary task, or their interaction was found on accuracy in the NCT, all *p* > 0.305.

3 To check whether this global effect becomes also evident for only uninterrupted trials, we ran another analysis while just considering the uninterrupted trials, resulting in a 3 (frequency condition) × 2 (target presence) ANOVA. This analysis confirmed the global effect of interruption frequency, with a significant main effect of frequency condition, *F*(2, 147) = 4.961, *p* = 0.008, η*_*p*_*^2^ = 0.063. Pairwise comparisons with Bonferroni-corrected p-values showed faster responses in uninterrupted trials in the high (2,073 ms) than in the low (2,402 ms, *p* = 0.009) frequency condition and no significant differences (*p*s > 0.064) to the mid frequency (2,402 ms) condition. In this ANOVA, the main effect of target was also significant (*p* < 0.001) and the interaction non-significant (*p* = 0.656), in line with the overall analysis.

4 PrBIC(H_0_| D) is an estimation of the posterior probability for the null hypothesis and can be calculated from the sums of squares of a traditional null hypothesis significance testing ANOVA. Larger values indicate evidence for the null hypothesis, with values between 0.75 and 0.95 being typically considered as “positive” evidence for the null (see e.g., Table 3 of Wagenmakers, 2007).
